# Incidence and Risk Factors for Acute Kidney Injury after Allogeneic Stem Cell Transplantation: A Prospective Study

**DOI:** 10.3390/biomedicines10020262

**Published:** 2022-01-25

**Authors:** Andreea Andronesi, Bogdan Sorohan, Andreea Burcea, Lavinia Lipan, Cristina Stanescu, Oana Craciun, Laura Stefan, Adela Ranete, Zsofia Varady, Oana Ungureanu, Gabriela Lupusoru, Gabriela Agrigoroaei, Danut Andronesi, Luminita Iliuta, Bogdan Obrisca, Alina Tanase

**Affiliations:** 1 Department of Nephrology, Carol Davila University of Medicine and Pharmacy, 020021 Bucharest, Romania; bogdan.sorohan@drd.umfcd.ro (B.S.); gabriela.lupusoru@umfcd.ro (G.L.); gabriela.agrigoroaei@rez.umfcd.ro (G.A.); bogdan.obrisca@drd.umfcd.ro (B.O.); 2 Nephrology Department, Fundeni Clinical Institute, 022328 Bucharest, Romania; andreea.burcea@rez.umfcd.ro (A.B.); cristina.cristache@rez.umfcd.ro (C.S.); oana-catalina.ion@drd.umfcd.ro (O.U.); 3 Bone Marrow Transplant Department, Fundeni Clinical Institute, 022328 Bucharest, Romania; lavinia.lipan@icfundeni.ro (L.L.); oana.craciun@icfundeni.ro (O.C.); laura.stefan@icfundeni.ro (L.S.); adela.ranete@icfundeni.ro (A.R.); zsofia.varady@rndvcsh.ro (Z.V.); alina.tanase@icfundeni.ro (A.T.); 4 Department of General Surgery and Liver Transplant, Fundeni Clinical Institute, 022328 Bucharest, Romania; dan_andronesi@yahoo.com; 5 Department of Biostatistics, Marketing and Medical Technology, Carol Davila University of Medicine and Pharmacy, 020021 Bucharest, Romania; luminita.iliuta@umfcd.ro

**Keywords:** acute kidney injury, stem cell transplantation, calcineurin inhibitors, myeloablative

## Abstract

(1) Background: Acute kidney injury (AKI) is a serious complication of hematopoietic stem cell transplantation (HSCT). (2) Methods: The aim was to identify the incidence, severity, and risk factors for AKI during the first 100 days after allo-HSCT; we performed a prospective observational study on 135 consecutive patients. (3) Results: The mean age was 38.3 ± 11.9 years (50.6% females), AKI developed in 93 patients (68.9%), the median time of appearance was 28 days, and the mean serum creatinine at the time of AKI was 1.8 ± 0.8 mg/dL. A total of 36 (38.7%) patients developed stage 1 AKI, 33 (35.5%) patients developed stage 2, and 24 (25.8%) patients developed stage 3; eight (8.6%) patients required temporary hemodialysis, and the mortality rate in these patients was 87.5%. Death was twice as frequent in the AKI subgroup, without statistical significance. Cyclosporine overdose (HR = 2.36, 95% CI: 1.45–3.85, *p* = 0.001), tacrolimus overdose (HR = 4.72, 95% CI: 2.22–10.01, *p* < 0.001), acute graft-versus-host disease (aGVHD) (HR = 1.96, 95% CI: 1.13–3.40, *p* = 0.01), and CRP level (HR = 1.009, 95% CI: 1.007–1.10, *p* < 0.001) were independent risk factors for AKI. Sepsis (HR = 5.37, 95% CI: 1.75–16.48, *p* = 0.003) and sinusoidal obstruction syndrome (HR = 5.10, 95% CI: 2.02–12.85, *p* = 0.001) were found as independent risk factors for AKI stage 3. (4) Conclusions: AKI occurs with high incidence and increased severity after allo-HSCT. Careful monitoring of calcineurin inhibitors and proper management of sepsis may reduce this risk.

## 1. Introduction

Hematopoietic stem cell transplantation (HSCT) is a complex therapy with curative potential for an expanding array of diseases, both malignant and non-malignant. Data from the literature indicate that a milestone of 1.5 million HCSTs performed worldwide was reached in 2019 [1]. The rising number of allogeneic HSCTs (allo-HSCT) reported annually is paralleled by improved outcomes. Acute kidney injury (AKI) is a frequent complication among patients undergoing allogeneic HSCT. A recent, large cohort study found a 64% incidence during the first 100 days following the procedure, demonstrating a higher non-relapse mortality in the group experiencing kidney dysfunction [2].

Although the prognosis of patients undergoing HSCT has improved in recent years, a meta-analysis of 36 cohort studies found that AKI incidence after HSCT remained high throughout the years, affecting more than half of the patients [3]. As such, a proper assessment of the risk factors associated with AKI occurrence in the HSCT population is of extreme importance, since HSCT patients’ prognosis may be additionally improved by addressing the modifiable ones.

Although several studies approached the subject of AKI following HSCT, most of them were performed in a retrospective manner [3,4,5]. As well, the literature provides inconsistent evidence on the relationship between AKI and calcineurin inhibitors (CNI) overdosing [4,5].

## 2. Materials and Methods

### 2.1. Study Design

We performed a prospective observational study on a cohort of 135 consecutive patients with various hematologic conditions, both malignant and non-malignant, requiring allo-HSCT. Patients were followed for 100 days after this procedure. The aim of our study was to observe the occurrence of AKI and the risk factors for this event after allo-HSCT.

### 2.2. Hematopoietic Stem Cell Transplantation

HSCT eligibility was based on the following criteria: diagnosis of hematological diseases with standard indications of allo-HSCT, age between 18 and 70 years, no significant major organ failure (heart, lungs, liver), estimated glomerular filtration rate (eGFR) of at least 55 mL/min/1.73 m^2^ of body surface area (using CKD-EPI formula prior to transplantation), no uncontrolled diabetes mellitus, no active infections, ECOG (Eastern Oncology Cooperative Group) [6] performance status ≤2, and prior psychiatric assessment. The preferred stem cell source for HSCT for our patients was peripheral blood and a minority of the procedures were performed using bone marrow. Stem cells harvesting and myeloablative or non-myeloablative conditioning regimens were used according to center protocols and depending on patients’ age and comorbidities (estimated by Hematopoietic cell transplantation—specific comorbidity index HCT-CI) [7]. Regimens using total body irradiation (TBI) are rarely used in our institute due to economic reasons. For all the patients who received TBI, individualized shields were used for protecting critical organs like lungs and kidneys. All patients received graft-versus-host disease (GVHD) prophylaxis using either cyclosporine or tacrolimus (with trough levels of at least 100 ng/mL, optimum 200–300 ng/mL for cyclosporine, and at least 5 ng/mL, optimum 5–10 ng/mL for tacrolimus). All patients received anti-infective prophylactic therapy with quinolones (ciprofloxacin or levofloxacin), acyclovir and azoles (posaconazole or voriconazole). Broad spectrum antibiotics were used according to the institutional protocol in the event of febrile neutropenia.

### 2.3. Collected Data and Follow-Up

Data collected included gender, age, type and length of underlying hematologic disease, type of conditioning regimens (myeloablative or non- myeloablative), donor type (siblings, haploidentical, unrelated 10/10 and 9/10 HLA-matched), stem cell source (bone marrow or peripheral stem blood cells), comorbidities (HCT-CI score), aplasia recovery day, hospital length stay, and complications with potential to affect kidney function: sinusoidal obstruction syndrome (SOS), GVHD, thrombotic microangiopathy (TMA), sepsis, and febrile neutropenia. The need for temporary dialysis in patients who developed AKI and death within the first 100 days after transplant were also recorded.

Comprehensive chemistry panels were performed during the follow up period, including CRP level and serum creatinine for AKI diagnosis and staging. eGFR [8] was also evaluated after 90 days following AKI episode for surviving patients who developed this complication. Use of other concomitant nephrotoxic substances was noted. Cyclosporine and tacrolimus overdoses were interpreted as trough levels above 300 ng/mL and 15 ng/mL respectively.

### 2.4. Definitions

AKI definition and staging were done according to KDIGO clinical practice guidelines [9]. Regarding the stage used in analysis, we chose the highest AKI stage occurring during the first 100 days after HSCT. Renal recovery was interpreted as a decline in serum creatinine (SCr) to less or within 0.3 mg/dL above the baseline value obtained 3 months after the AKI event. SOS was defined according to the definition used by the British Society of Hematology [10], while for the diagnosis and staging of GVHD, the National Institutes of Health Report was used [11]. We defined mixed GVHD (mGVHD) as concomitant involvement of skin and gastrointestinal system. TMA was diagnosed according to criteria proposed by the International Working Group [12]. Sepsis was defined according to the Sepsis-3 International Consensus [13].

### 2.5. Statistical Analysis

Variables are reported as follows: percentages for categorical data, mean ± standard deviation for normally distributed data, and median with interquartile range (IQR) for continuous nonparametric data. Chi-square test and Fisher’s exact test were used as appropriate to evaluate the statistical differences between groups for categorical variables. Student’s *t*-test and Mann–Whitney U test were used to analyze the differences between groups for continuous parametric and non-parametric variables, respectively. To identify the risk factors for AKI, we used Cox proportional hazard analysis. In the Cox univariate analysis, we included variables with a *p*-values less than 0.15 at groups comparison. In the Cox multivariate analysis model, we introduced all variables from the univariate. As well, in the latter process a stepwise backward elimination process was applied. The cumulative incidence of AKI was represented as Kaplan–Meier curves. The comparison between hazard distributions was determined by log-rank test. A *p*-value < 0.05 was considered statistically significant. Statistical analysis was performed using IBM SPSS Statistics, Version 26 (SPSS Inc., Chicago, IL, USA) and STATA Version 14.

## 3. Results

### 3.1. Patients’ Characteristics and Incidence of Acute Kidney Injury

Clinical, laboratory, transplant features, and complications of the 135 patients with HSCT are reported in Table 1. The mean age at the time of transplant was 38.3 ± 11.9 years; 50.6% were females and the most common underlying hematologic disease was AML/MDS (56.3%). Peripheral blood was the main donor cell source (97%) and in 65.2% of patients, a myeloablative conditioning regimen was performed. The mean eGFR at the moment of transplant was 110.8 ± 18.5 mL/min/1.73m^2^. Regarding complications after HSCT, fever was the most frequent (54.1%), followed by GVHD (34.1%) and TMA (28.1%). Skin and gastrointestinal GVHD type were the most commonly found (14.1%), and most patients had grade I (11.1%) and II (14.8%) GVHD. Death was reported in 19 out of 135 patients (14.1%) and the median time of death was 33 days (IQR: 20–65).

**Figure 1:**
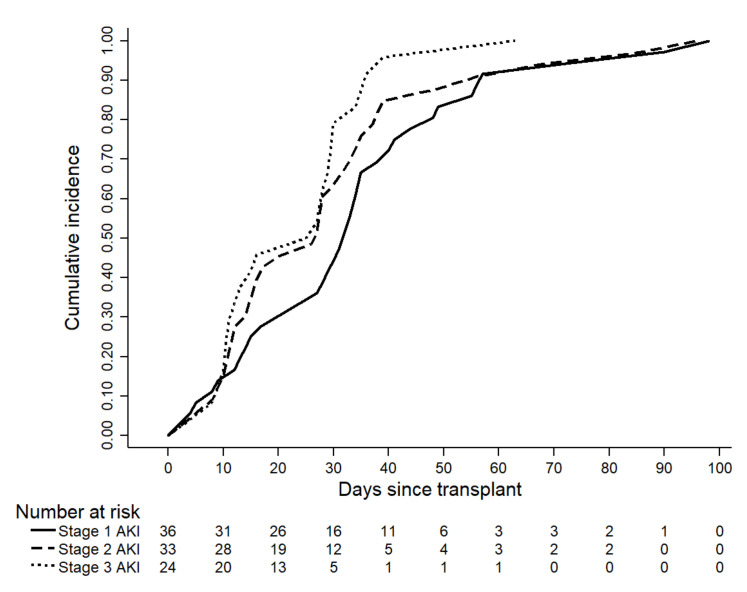
Kaplan–Meier curves for the cumulative incidence of AKI according to its stage.

AKI developed in 93 patients (68.9%) in the first 100 days after HSCT. Among these patients, AKI occurred in 56 patients (41.4%) within the first 30 days. The median time of AKI appearance was 28 days (IQR: 12.5–35.5) and the mean serum creatinine at the time of AKI was 1.8 ± 0.8 mg/dL. Of the 93 patients with AKI, 36 (38.7%) had stage 1 AKI, 33 (35.5%) had stage 2, and 24 (25.8%) had stage 3. Eight (8.6%) patients required renal replacement therapy (RRT). The cumulative probability of AKI according to AKI stage was higher for stage 3 than stage 2 and stage 1 at 30 and 60 days after HSCT (Figure 1).

### 3.2. Comparison between AKI and Non-AKI Subgroups

Patients from the AKI subgroup had undergone significantly more myeloablative conditioning regimens (73.1% vs. 47.6%, *p* = 0.004), TBI (11.8% vs. 2.4%, *p* = 0.04), had more frequent CsA (34.4% vs. 2.4%, *p* < 0.001) and TAC (9.7% vs. 0%, *p* = 0.008) overdosage, and they received fewer fludarabine-based regimens (51.5% vs. 69%, *p* = 0.05) and MTX (31.2% vs. 85.7%, *p* < 0.001). As well, those patients had complications more often, including SOS (21.5% vs. 4.8%, *p* = 0.01), mGVHD (18.3% vs. 4.8%, *p* = 0.004), TMA (34.3% vs. 14.3%, *p* = 0.01), and a significantly higher baseline level of CRP (24.3 (IQR: 2.9–123.5) vs. 10 (IQR: 3–21), *p* = 0.02). Death was approximately twice as frequent in the AKI subgroup, but without statistical significance. There were no significant differences between AKI and non-AKI patients in terms of demographic data, underlying hematological disease, donor data, comorbidities, and kidney function at the time of HSCT (Table 1).

### 3.3. Risk Factors for Acute Kidney Injury

Cox regression analysis was used to assess the risk factors associated with AKI within 100 days of HSCT. Univariate Cox regression model included all variables with *p*-value < 0.15 at subgroup comparison (Table 2). By univariate Cox regression analysis, male gender (HR = 1.25, 95% CI = 0.83–1.88, *p* = 0.27), ALL (HR = 1.37, 95% CI = 0.88–2.13, *p* = 0.16), and GVHD grade I (HR = 0.75, 95% CI = 0.37–1.52, *p* = 0.43) and II (HR = 1.39, 95% CI = 0.78–2.45, *p* = 0.25) were the only variables that were not statistically significant. By multivariate Cox regression analysis, CsA overdose (HR = 2.36, 95% CI = 1.45–3.85, *p* = 0.001), TAC overdose (HR = 4.72, 95% CI = 2.22–10.01, *p* = 0.001), acute GVHD (HR = 1.96, 95% CI = 1.13–3.40, *p* = 0.01), and CRP serum level (HR = 1.009, 95% CI = 1.007–1.10, *p* < 0.001) were found as independent risk factors for AKI within 100 days after HSCT. Male gender (HR = 1.46, 95% CI = 0.96–2.23, *p* = 0.07) and ALL (HR = 1.59, 95% CI = 0.98–2.58, *p* = 0.05) presented only a trend of significance (Table 2).

**Figure 2:**
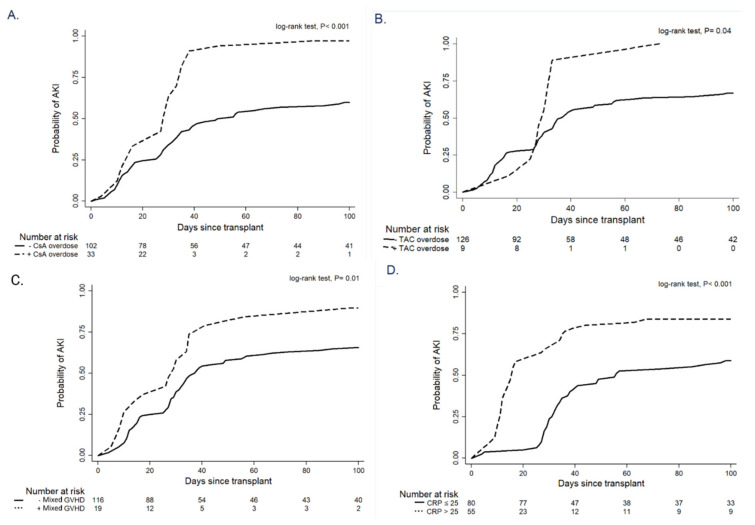
Kaplan–Meier curves showing cumulative risk of AKI according to the presence of CsA overdosage (**A**), TAC overdosage (**B**), mixed GVHD (**C**) and CRP value (**D**).

A Kaplan–Meier analysis showed that cumulative probability of AKI in the first 100 days after HSCT was higher in patients with CsA overdose (96.9% vs. 59.8%, *p* < 0.001), TAC overdose (100% vs. 66.7%, *p* = 0.04), presence of acute GVHD (89.5% vs. 65.5%, *p* = 0.01) and CRP level >25 mg/L (83.6% vs. 58.7%, *p* < 0.001) (Figure 2).

Characteristics of patients with AKI stage 3 are presented in Table 3. We observed that patients with ALL developed frequently significant AKI stage 3 after allo-HSCT (50% vs. 21.6%, *p* = 0.007). Also, patients from the severe AKI subgroup significantly often received myeloablative conditioning regimens (87.5% vs. 60.4%, *p* = 0.01), TBI (20.8% vs. 6.3%, *p* = 0.03), and fludarabine-based regimens (29.2% vs. 63.1%, *p* = 0.002) and less often MTX (25% vs. 53.2%, *p* = 0.01). Moreover, these patients more often developed complications (SOS (50% vs. 9%, *p* < 0.001), grade 2 and 3 GVHD (25% vs. 12.6% for grade 2 GVHD, 33.3% vs. 2.7% for grade 3 GVHD, respectively, *p* < 0.001), TMA (50% vs. 23.4%, *p* = 0.009), fever (87.5% vs. 46.8%, *p* < 0.001)), and had a significantly higher baseline level of CRP (83.3 (IQR: 22.9–218.6) vs. 7.8 (IQR: 2.7–46), *p* < 0.001).

By univariate Cox regression analysis all variables included in the model, but not grade 1 GVHD (HR = 1.34; 95%CI = 0.36–1.97, *p* = 0.55) were significantly associated with AKI stage 3. In multivariate Cox regression analysis, sepsis (HR = 5.37, 95% CI = 1.75–16.48, *p* = 0.003) and SOS (HR = 5.10, 95% CI = 2.02–12.85, *p* = 0.001) were independent risk factors associated with AKI stage 3, while ALL (HR = 1.59, 95% CI = 0.98–2.58, *p* = 0.05) had a trend of significance (Table 4).

## 4. Discussion

Allogeneic HSCT is used to treat both malignant and non-malignant hematologic diseases, the most common indications being leukemia and lymphoma [14,15]. These also represent more than 90% of the conditions for which subjects in our study received allo-HSCT. AKI incidence depends on the type of transplant (allogeneic transplant is associated with a higher occurrence of kidney injury versus autologous transplant), conditioning regimen (myeloablative chemotherapy affects kidney function to a greater extent compared to non-myeloablative regimens), and AKI definition [16]. In our cohort, the AKI incidence during the first 100 days after allo-HSCT was 68.9%. In a previous study performed by our team, we found a 10% incidence of AKI in the first 30 days following autologous stem cell transplantation performed in patients with multiple myeloma [17], whereas in the present study we had an incidence of 41.4% in the first month. This high risk of AKI could be attributed to the severity of myeloablative regimens. A study of 201 cases of allogeneic transplant reported an AKI incidence of 62–66% for the group that received myeloablative conditioning and 40–48% in the non-myeloablative category. In each group three patients required dialysis and all of them died in the follow-up period [18]. A similar study reported an AKI incidence of 65.1% among patients treated with allogeneic transplant and a mortality of 9.7% [19]. Although the incidence of AKI was relatively high, only eight patients from our study required emergency hemodialysis. However, the death rate among patients requiring RRT was very high—seven out of these eight patients (87.5%) died. All AKI survivors completely recovered their renal function at 3 months after the AKI episode, irrespective of its staging.

Prophylaxis against GVHD is most commonly achieved using CNI. These immunosuppressive agents are well known for their nephrotoxicity. The acute type of injury is thought to rather be a functional impairment as a result of afferent arteriolar vasoconstriction, in contrast with the chronic insult that is associated with histological lesions. Consequently, in the acute setting, the reduction in GFR is reversible with dose lowering. Elements that play a role in CNI induced acute vascular dysfunction showed a decrease in vasodilator factors (nitric oxide, prostaglandins), as well as an increase in vasoconstrictors (endothelin, thromboxane) and activation of both the sympathetic nervous system and the renin-angiotensin system [20]. Moreover, they have been linked to an increased liability for developing TMA in the HSCT population [21]. We found that CsA and TAC overdose were independent risk factors for AKI. The published studies provided contradictory results regarding the relationship between CNI overdose and increased risk for AKI after allo-HSCT. A study that examined the association between blood levels of CsA and incidence of AKI found that an increase in the CNI level did not correlate with a higher risk of kidney injury after myeloablative HSCT [4]. With respect to the reduced-intensity conditioning experience, a single-center study of 188 cases reported CNI to be the main cause of AKI in these patients. There was again no correlation with plasma levels of CsA, but the values did not exceed 300 ng/mL in that study [5]. Nevertheless, more than 35 years ago, Kennedy et al. demonstrated that a CsA trough level greater than 250 ng/mL was associated with kidney injury in patients receiving this drug for GVHD prophylaxis [22]. A possible explanation for the lack of abundant information concerning CNI overdose and AKI after bone marrow transplantation in recent literature could be the continuous adaptation of doses in response to an ascending trend of serum creatinine, as nephrotoxicity is reversible. Moreover, although the monitoring of trough levels of CNI is nowadays a standard of care, overdosage is not so rare due to high intrapatient variability of CNI metabolism and because of many drug-to-drug interactions, including some drugs which are usually used in HSCT, such as azoles.

Multivariate analysis indicated mGVHD to be an independent risk factor for AKI. Acute GVHD is an immune disorder in which alloreactive T cells from the donor attack healthy structures in the recipient, most frequently the skin, liver, and gastrointestinal tract. Among these three organs, gastrointestinal involvement represents the greatest source of GVHD-related mortality, as injury to the gut mucosal barrier can lead to complications such as intractable vomiting and voluminous diarrhea, up to several liters per day [23]. The resulting extracellular volume depletion will therefore be an important cause of prerenal AKI in these patients [24]. Moreover, an intact intestinal barrier is essential in preventing bacteria and endotoxins to enter the bloodstream, so these patients are more prone to develop gastrointestinal sepsis with associated AKI. Acute GVHD has in fact been proven to independently raise the risk for AKI events both in the patients who received myeloablative, as well as non-myeloablative conditioning regimens [25]. The hypothesis of the kidney being a target organ after allo-HSCT has been explored in murine models. Sadeghi et al. demonstrated that various genes involved in antigen presentation and immune response were overexpressed in the kidneys of acute GVHD mice. Moreover, histopathologic staining showed T cell invasion in the kidney after developing GVHD [26,27]. Apart from dehydration, cytokine and immune-related injury, and acute GVHD treatment itself with high-dose corticosteroids, can also predispose to AKI occurrence by increasing the risk of CMV reactivation [25].

ALL was the underlying hematologic disease for 36 patients in our study and we found a striking association between ALL and the risk for AKI—29 out of these 36 patients developed AKI, and ALL approached statistical significance as a risk factor for AKI in multivariate analysis. A recent, large cohort study examining AKI occurrence across various types of malignancies identified an increased risk of AKI in patients with leukemia that had undergone HSCT [28]. A novel study found that ALL patients (compared to AML) had a higher risk of developing transplant-associated TMA, presumably due to the more frequent use of TBI regimens in this group of patients [29]. Considering that the kidneys are often affected in the event of TMA, this could be a possible explanation for our findings.

Fludarabine-based regimens proved to be a protective factor against AKI in our patients. This observation was also made in a cohort of patients treated for AML or MDS— the patients who received a non-fludarabine-based chemotherapy regimen had an independently higher risk of developing AKI [30]. A possible explanation could be that they are less harmful on the gastrointestinal mucosa, thus preventing a series of complications that could further predispose to AKI.

TBI is a component of the myeloablative conditioning regimen in selected patients. This was the case for 12 participants in our study, and 11 out of these 12 patients developed AKI within 100 days following HSCT. The susceptibility of kidneys to irradiation is a well-defined concept, but they are not classified among early-responsive tissues, as radiation nephropathy mainly occurs approximately 1–1.5 years after irradiation [31]. For this reason, renal shielding should be taken into consideration in these patients [32]. TBI has also been labeled as a risk factor for TMA and SOS, complications that can independently predispose to AKI [21,33,34]. Therefore, this could be a possible explanation for some of the early renal dysfunction cases detected in our cohort, as 6 out of the 12 patients that had received TBI also developed TMA, SOS, or both.

A complication frequently arising in HSCT patients is TMA, as various factors including CNI use, TBI, and GVHD may contribute to abnormal endothelial cell activation. The incidence in the literature fluctuates between 0.5 and 76%, mainly as a result of inconsistent diagnostic criteria and different levels of awareness [35]. Kidneys are generally considered to be the main site of microangiopathy, with consequences varying from proteinuria to AKI and CKD [36]. In our cohort, TMA was a frequent complication after HSCT, documented in almost one fourth of the patients; over 80% of them also developed AKI. In a recent study that evaluated more than 23,000 allo-HSCT recipients, Epperla et al. noted a significantly greater risk of RRT and mortality in patients who developed TMA [29].

Another complication targeting the endothelial cells in HSCT patients is SOS, formerly known as hepatic veno-occlusive disease (VOD) [37]. We found a 14% incidence of SOS, similar to that reported in the literature [38], but the SOS cases were significantly higher in the AKI group—20 out of the 22 patients with SOS also developed AKI. Indeed, this rare complication has been shown to be associated with AKI and it is considered to be a variant of hepatorenal syndrome, considering the fluid retention and the relative hypoperfusion of the kidneys that are part of the clinical picture [16]. A recent study that included over 200 cases of SOS observed that during the 7 days prior to this diagnosis, there were significantly more cases of AKI compared to the control group, indicating that renal hypoperfusion might be an early event in SOS [38].

We found a significant difference in CRP levels between the AKI and non-AKI subgroups and CRP level was found to be an independent risk for AKI after allo-HSCT. This close association between high CRP level and renal dysfunction was demonstrated in many cohorts of patients with contrast-induced nephropathy and post-operative AKI, mainly after cardiac surgery, but also after non-cardiac interventions [39]. Studies in the last decades emphasized that CRP mediates biological activities [40], including the pathogenesis and progression of AKI. For example, a group that used CRP transgenic mice found that this molecule promotes AKI through impaired tubular epithelial cell regeneration, by induction of G1 cell cycle arrest. They also noted that, after inducing inchemia/reperfusion AKI, CRP played a role in increased epithelial cell necrosis, thus exacerbating the tissue damage [41]. Another animal model study showed that mice with a higher level of human CRP developed inflammation faster, particularly due to increased expression of proinflammatory mediators, associated among others with endogenous renal CRP [42]. The association observed in our study could therefore originate in both the underlying inflammation accompanying complications predisposing to AKI, especially sepsis, but also the intrinsic capacity of CRP to exacerbate local inflammation in the kidney. Sepsis is considered the most important cause of AKI in critically ill patients [43], and it was the most significant independent risk factor for stage 3 AKI in our cohort. Sepsis is a state of decreased effective kidney perfusion due to systemic vasodilation and increased capillary permeability with extravascular leak due to cytokine storm, with prerenal AKI, but it also causes intrinsic AKI due to endothelial damage with microthrombi formation and a maladaptive response to the energetic stress of tubular epithelial cells [43,44].

Our study has several limitations. It included patients from a single center, so our conclusions need to be further validated in larger cohort studies. Moreover, the use of creatinine rise as an indicator of kidney injury may be less reliable compared to other biomarkers. However, our research has some important strengths, especially regarding the prospective nature of the study and the significant number of AKI events, factors which increase the statistical power to identify the most important risk factors for AKI occurrence.

## 5. Conclusions

Our study showed that AKI is a frequent complication after allo-HSCT and the predisposing factors are often essential components of the complex process of bone marrow transplantation. Early identification of patients at risk of developing kidney injury, together with proper hydration, avoidance of additional nephrotoxins exposure and the close monitoring of calcineurin inhibitor blood levels may be the key to further improve outcomes. Future studies are needed to shed light on the pathophysiology of AKI in allo-HSCT and the optimal management strategies.

## Figures and Tables

**Table 1:** Clinical, laboratory, transplant characteristics, and complications of the study patients.

	Overall (N = 135)	No AKI (N = 42)	AKI (N = 93)	*p*-Value
Demographic data				
Age at transplant (mean, years)	38.3 ± 11.9	39.7 ± 11.6	37.6 ± 12.1	0.34
Male gender (%)	64 (47.4 %)	16 (38.1%)	48 (51.6%)	0.14
Underlying hematologic disease (%)				0.15
AML/MDS ALL Lymphoma CML Aplastic anemia	76 (56.3%) 36 (26.7%) 13 (9.6%) 4 (3.0%) 6 (4.4%)	25 (59.5%) 7 (16.7%) 4 (9.5%) 2 (4.8%) 4 (9.5%)	51 (54.8%) 29 (31.2%) 9 (9.6%) 2 (2.2%) 2 (2.2%)	
Hematologic disease length (median, months)	9 (6–15)	7 (5–14.2)	10 (6–16.5)	0.17
Donor type (%)				0.95
Siblings Haploidentical 10/10 9/10	93 (68.9%) 6 (4.4%) 23 (17.0%) 13 (9.7%)	30 (71.4%) 2 (4.8%) 6 (14.3%) 4 (9.5%)	63 (67.7%) 4 (4.3%) 17 (18.3%) 9 (9.7%)	
Donor cell source (%)				0.42
Peripheral blood Bone marrow	131 (97%) 4 (3%)	40 (95.2%) 2 (4.8%)	91 (97.8%) 2 (2.2%)	
Conditioning type (%)				0.004 ^a^
Myeloablative Non-myeloablative	88 (65.2%) 47 (34.8%)	20 (47.6%) 22 (52.4%)	68 (73.1%) 25 (26.9%)	
TBI (%)	12 (8.9%)	1 (2.4%)	11 (11.8%)	0.04 ^a^
Fludarabine-based regimen (%)	77 (57.0%)	29 (69.0%)	48 (51.6%)	0.05
Aplasia recovery period (mean, days)	18.2 ± 3.5	18.6 ± 3.8	17.9 ± 3.3	0.30
Hospital length of stay (mean, days)	24.9 ± 9.9	24.4 ± 4.8	25.2 ± 11.6	0.67
Comorbidities (%)				
Hypertension Type 2 DM HBV infection HCV infection	4 (3.0%) 4 (3.0%) 20 (14.8%) 3 (2.2%)	1 (2.4%) 1 (2.4%) 7 (16.7%) 1 (2.4%)	3 (3.2%) 3 (3.2%) 13 (14.0%) 2 (2.2%)	0.78 0.78 0.68 0.93
Kidney function at transplantation				
Serum creatinine (mean, mg/dL) eGFR (mean, mL/min)	0.7 ± 0.1 110.8 ± 18.5	0.71 ± 0.1 109.8 ± 16.4	0.73 ± 0.1 111.3 ± 19.4	0.47 0.66
Complications (%)				
SOS (%)	22 (16.3%)	2 (4.8%)	20 (21.5%)	0.01 ^a^
GVHD (%)	46 (34.1%)	11 (26.2%)	35 (37.6%)	0.19
Mixed GVHD (%)	19 (14.1%)	2 (4.8%)	17 (18.3%)	0.004 ^a^
GVHD grading (%)				0.10
1	15 (11.1%)	6 (14.3%)	9 (9.7%)	
2	20 (14.8%)	5 (11.9%)	15 (16.1%)	
3	11 (8.1%)	0 (0%)	11 (11.8%)	
TMA (%)	38 (28.1%)	6 (14.3%)	32 (34.4%)	0.01 ^a^
Fever (%)	73 (54.1%)	19 (45.2%)	54 (58.1%)	0.16
Sepsis (%)	24 (17.8%)	9 (21.4%)	9 (21.4%)	0.45
Death (%)	19 (14.1%)	3 (7.1%)	16 (17.25)	0.12
CRP serum level (median, mg/L)	14.2 (3.0–80.0)	10 (3–21)	24.3 (2.9–123.5)	0.02 ^a^
Nephrotoxic drugs				
CNI type (%)				0.61
Cyclosporine	109 (80.7%)	35 (83.3%)	74 (79.6%)	
Tacrolimus	26 (19.3%)	7 (16.7%)	19 (20.4%)	
CsA overdosage (%)	33 (24.4%)	1 (2.4%)	32 (34.4%)	<0.001 ^a^
TAC overdosage (%)	9 (6.7%)	0 (0%)	9 (9.7%)	0.008 ^a^
Contrast media (%)	12 (8.9%)	5 (11.9%)	7 (7.5%)	0.41
MTX (%)	65 (48.1%)	36 (85.7%)	29 (31.2%)	<0.001 ^a^
Antibiotics (%)	40 (29.6%)	14 (33.3%)	26 (28.0%)	0.52

Chi-square test and Fisher’s exact test—categorical variables; Student’s *t*-test—continuous normally distributed variables; Mann–Whitney U test—continuous nonparametric variables. N—Number; AKI—Acute kidney injury; AML—Acute myeloid leukemia; MDS—myelodysplastic syndrome; ALL—Acute lymphoblastic leukemia; CML—Chronic myeloid leukemia; TBI—total body irradiation; DM—diabetes mellitus; HBV—hepatitis B virus; HCV—hepatitis C virus; eGFR—estimated glomerular filtration rate; SOS—Sinusoidal obstruction syndrome; GVHD—graft versus host disease; mGVHD—mixed graft versus host disease; TMA—thrombotic microangiopathy; CRP—C reactive protein; CNI—Calcineurin inhibitors; CsA—Cyclosporin; TAC—Tacrolimus; MTX—Methotrexate; ^a^ statistically significant.

**Table 2:** Univariate and multivariate Cox regression analysis for evaluation of risk factors associated with AKI in the first 100 days after HSCT.

	Univariate Cox Regression Analysis			Multivariate Cox Regression Analysis		
Variables	HR	95% CI	*p*-Value	HR	95% CI	*p*-Value
Male gender	1.25	0.83–1.88	0.27	1.46	0.96–2.23	0.07
Acute lymphoblastic leukemia	1.37	0.88–2.13	0.16	1.59	0.98–2.58	0.05
Myeloablative conditioning regimens	2.27	1.43–3.60	<0.001 ^a^			
TBI	2.49	1.31–4.72	0.005 ^a^			
Fludarabine	0.57	0.38–0.86	0.008 ^a^			
MTX	0.42	0.26–0.66	<0.001 ^a^			
CsA overdose	2.78	1.78–4.35	<0.001 ^a^	2.36	1.45–3.85	0.001 ^a^
TAC overdose	2.02	1.01–4.06	0.04 ^a^	4.72	2.22–10.01	<0.001 ^a^
mGVHD	1.89	1.11–3.21	0.01 ^a^	1.96	1.13–3.40	0.01 ^a^
GVHD grading						
I	0.75	0.37–1.52	0.43			
II	1.39	0.78–2.45	0.25			
III	2.00	1.05–3.82	0.03 ^a^			
Sinusoidal obstruction syndrome	2.42	1.46–4.00	0.001 ^a^			
Thrombotic microangiopathy	1.77	1.15–2.72	0.009 ^a^			
CRP level	1.008	1.006–1.010	<0.001 ^a^	1.009	1.007–1.10	<0.001 ^a^

HR—hazard ratio; CI—confidence interval; TBI—total body irradiation; MTX—methotrexate; CsA—cyclosporine; TAC—tacrolimus; mGVHD—mixed graft versus host disease; CRP—C reactive protein. Multivariate Cox regression analysis: variables remained in the final step after backward stepwise selection: male gender, acute lymphoblastic leukemia, CsA overdose, TAC overdose, acute GVHD, CRP level; ^a^ statistically significant.

**Table 3:** Clinical, laboratory, transplant characteristics, and complications of the study patients according to AKI stage 3.

	No AKI Stage 3 (N = 111)	AKI Stage 3 (N = 24)	*p*-Value
Demographic data			
Age at transplant (mean, years)	38.9 ± 11.9	35.5 ± 11.8	0.19
Male gender (%)	55 (49.5%)	9 (37.5%)	0.28
Underlying hematologic disease (%)			0.007
AML/MDS ALL Lymphoma CML Aplastic anemia	69 (62.2%) 24 (21.6%) 10 (9%) 2 (1.8%) 6 (5.4%)	7 (29.2%) 12 (50%) 3 (12.5%) 2 (8.3%) 0 (0%)	
Hematologic disease length (median, months)	8 (6–14)	10.5 (6.2–27.2)	0.21
Donor type (%)			0.25
Related Unrelated haploidentical 10/10 9/10	73 (65.8%) 5 (4.5%) 20 (18%) 13 (11.7%)	20 (83.3%) 1 (4.2%) 3 (12.5%) 0 (0%)	
Donor cell source (%)			1
Peripheral blood Bone marrow	107 (96.4%) 4 (3.6%)	111 (100%) 0 (0%)	
Conditioning type (%)			0.01
Myeloablative Non-myeloablative	67 (60.4%) 44 (39.6%)	21 (87.5%) 3 (2.5%)	
TBI (%)	7 (6.3%)	5 (20.8%)	0.03
Fludarabine based regimen (%)	70 (63.1%)	7 (29.2%)	0.002
Aplasia recovery period (mean, days)	18.3 ± 3.5	17.3 ± 3.6	0.20
Hospital length of stay (mean, days)	24.9 ± 8.2	25.2 ± 16.0	0.25
Comorbidities (%)			
Hypertension	4 (3.6%)	0 (0%)	0.34
Type 2 DM	4 (3.6%)	0 (0%)	0.34
HBV infection	17 (15.3%)	3 (12.5%)	1
HCV infection	2 (1.8%)	1 (4.2%)	0.44
Kidney function at transplantation			
Serum creatinine (mean, mg/dL) eGFR (mean, mL/min)	0.7 ± 0.1 108.8 ± 17.8	0.6 ± 0.1 120 ± 18.9	0.001 0.006
Complications (%)			
SOS (%)	10 (9%)	12 (50%)	<0.001
GVHD (%)	32 (28.8%)	14 (58.3%)	0.006
mGVHD (%)	13 (11.7%)	6 (25%)	0.11
GVHD grading (%)			<0.001
1	15 (13.5%)	0 (0%)	
2	14 (12.6%)	6 (25%)	
3	3 (2.7%)	8 (33.3%)	
TMA (%)	26 (23.4%)	12 (50%)	0.009
Fever (%)	52 (46.8%)	21 (87.5%)	<0.001
Sepsis (%)	17 (15.3%)	7 (29.2)	0.10
Death (%)	13 (11.7%)	12 (50%)	<0.001
CRP serum level (median, mg/L)	7.8 (2.7–46)	83.3 (22.9–218.6)	<0.001
Nephrotoxic drugs			
CNI type (%)			0.56
Cyclosporine	88 (79.3%)	21 (87.5%)	
Tacrolimus	23 (20.7%)	3 (12.5%)	
CsA overdosage (%)	25 (22.5%)	8 (33.3%)	0.26
TAC overdosage (%)	7 (6.3%)	2 (8.3%)	0.71
Contrast media (%)	8 (7.2%)	4 (16.7%)	0.22
MTX (%)	59 (53.2%)	6 (25%)	0.01
Antibiotics (%)	30 (27%)	10 (41.7%)	0.15

Chi-square test and Fisher’s exact test—categorical variables; Student’s *t*-test—continuous normally distributed variables; Mann–Whitney U test—continuous non-parametric variables. N-Number; AKI—Acute kidney injury; AML–Acute myeloid leukemia; MDS–myelodysplastic syndrome; ALL–Acute lymphoblastic leukemia; CML–Chronic myeloid leukemia; TBI–total body irradiation; DM–diabetes mellitus; HBV–hepatitis B virus; HCV–hepatitis C virus; eGFR—estimated glomerular filtration rate; SOS–Sinusoidal obstruction syndrome; GVHD–graft versus host disease; mGVHD–mixed graft versus host disease; TMA–thrombotic microangiopathy; CRP–C reactive protein; CNI–Calcineurin inhibitors; CsA–Cyclosporin; TAC–Tacrolimus; MTX–Methotrexate; ^a^ statistically significant.

**Table 4:** Cox regression analysis for evaluation of risk factors associated with AKI stage 3 in the first 100 days after HSCT.

	Univariate Cox Regression Analysis			Multivariate Cox Regression Analysis		
Variables	HR	95% CI	*p*-Value	HR	95% CI	*p*-Value
Acute lymphoblastic leukemia	2.77	1.24–6.21	0.01 ^a^	1.59	0.98–2.58	0.05
Myeloablative conditioning regimens	2.27	1.43–3.60	<0.001 ^a^			
TBI	4.32	1.58–11.78	0.004 ^a^			
Fludarabine	0.23	0.09–0.56	0.001 ^a^	0.34	0.11–1.01	0.06
MTX	0.37	0.14–0.94	0.03 ^a^	0.30	0.10–1.10	0.32
Acute GVHD	2.86	1.27–6.44	0.01 ^a^	2.19	0.87–5.49	0.09
mGVHD	2.67	1.06–6.76	0.03 ^a^			
GVHD grading						
I	1.34	0.36–1.97	0.55			
II	3.12	1.13–8.61	0.02 ^a^			
III	8.06	3.17–20.47	<0.001 ^a^			
Sinusoidal obstruction syndrome	2.42	1.46–4.00	0.001 ^a^	5.10	2.02–12.85	0.001 ^a^
Thrombotic microangiopathy	1.77	1.15–2.72	0.009 ^a^			
Sepsis	2.58	1.06–6.25	0.03 ^a^	5.37	1.75–16.48	0.003 ^a^
Baseline creatinine	0.02	0.002–0.31	0.005 ^a^	0.05	0.003–1.10	0.06

HR–hazard ratio; CI–confidence interval; TBI–total body irradiation; MTX–methotrexate; mGVHD–mixed graft versus host disease; Multivariate Cox regression analysis—variables remained in the final step after backward stepwise selection: acute lymphoblastic leukemia, fludarabine, MTX, GVHD, sinusoidal obstruction syndrome, sepsis, baseline creatinine; ^a^ statistically significant.

## Data Availability

The data presented in this study are available on request from the corresponding author. The data are not publicly available due to privacy restrictions.
